# TRPs in Tox: Involvement of Transient Receptor Potential-Channels in Chemical-Induced Organ Toxicity—A Structured Review

**DOI:** 10.3390/cells7080098

**Published:** 2018-08-07

**Authors:** Dirk Steinritz, Bernhard Stenger, Alexander Dietrich, Thomas Gudermann, Tanja Popp

**Affiliations:** 1 Bundeswehr Institute of Pharmacology and Toxicology, 80937 Munich, Germany; bernhard.stenger@gmx.de (B.S.); tjjpopp@outlook.de (T.P.); 2 Walther-Straub-Institute of Pharmacology and Toxicology, Ludwig-Maximilians-Universität Munich, 80336 Munich, Germany; alexander.dietrich@lrz.uni-muenchen.de (A.D.); thomas.gudermann@lrz.uni-muenchen.de (T.G.)

**Keywords:** toxicology, TRP channels, organ toxicity, chemicals, pollutants, chemosensor

## Abstract

Chemicals can exhibit significant toxic properties. While for most compounds, unspecific cell damaging processes are assumed, a plethora of chemicals exhibit characteristic odors, suggesting a more specific interaction with the human body. During the last few years, G-protein-coupled receptors and especially chemosensory ion channels of the transient receptor potential family (TRP channels) were identified as defined targets for several chemicals. In some cases, TRP channels were suggested as being causal for toxicity. Therefore, these channels have moved into the spotlight of toxicological research. In this review, we screened available literature in PubMed that deals with the role of chemical-sensing TRP channels in specific organ systems. TRPA1, TRPM and TRPV channels were identified as essential chemosensors in the nervous system, the upper and lower airways, colon, pancreas, bladder, skin, the cardiovascular system, and the eyes. Regarding TRP channel subtypes, A1, M8, and V1 were found most frequently associated with toxicity. They are followed by V4, while other TRP channels (C1, C4, M5) are only less abundantly expressed in this context. Moreover, TRPA1, M8, V1 are co-expressed in most organs. This review summarizes organ-specific toxicological roles of TRP channels.

## 1. Introduction

Most compounds originating from chemical industry, either as main products, intermediates, or as pollutants, frequently exhibit toxic properties. Exposure can result in severe adverse health effects or even death. Although health and safety measures are mandatorily introduced, at least in industrial nations, accidents may occur at any time [1,2]. One example is the Bhopal incident in India on 3 December 1984, when more than 40 t of methyl isocyanate, a precursor in pesticide production, was released, killing at least 2500 people [3]. Another is the accidental release of butene from a cut gas-pipeline which then became inflamed, and resulted in the explosion of an adjacent ethylene pipeline at BASF Ludwigshafen Germany in October 2017 [1]. Other omnipresent chemicals are phosgene and chlorine, which are essential for a broad range of chemical products. Approximately 15 million tons of chlorine are produced annually only in the US [4]. In addition to the unintended release of such chemicals, a systematic misuse of chemicals in warfare or terror attacks occurred in the recent past [5]. The most common route of exposure is through inhalation. Ingestion, dermal, and ocular absorption represent other possible entry routes, but account only for a smaller proportion. Chemicals can exhibit characteristic odors like “rotten eggs” in the case of thiol-containing compounds, but also pleasant smells like the flower-scented Lewisite, a chemical warfare agent from World War I. Some chemicals are irritants that trigger protective reflexes such as burning sensations, lacrimation, or cough. These findings clearly indicate that chemicals do interact with the human sensory system. As defense mechanisms are regarded to be uniform, a close correlation to the chemical structure of the causing compound was not suggested. Thus, it was also assumed that the molecular processes regarding chemical sensing were also rather unspecific. However, research in recent years has clearly demonstrated that distinct G-protein-coupled taste and olfactory receptors as well as chemosensory ion channels of the transient receptor potential family (TRP channels)—are fundamentally involved in the molecular (patho)physiology with regard to chemical perception [6,7,8,9,10]. Especially, TRPA1 channels were found to be activated by various compounds of different origins, and at least for some chemicals, certain TRP channels were identified as molecular targets that mediate toxicity. Although the precise molecular mechanism of TRP channel activation has not been unraveled in all cases, these channels represent promising therapeutic targets to counteract chemical-induced toxicity. Therefore, it is not surprising that chemosensing TRP channels are regarded as central players, and are in the spotlight of today’s experimental, but also clinical pharmacological research [7,11].

**Figure 1:**
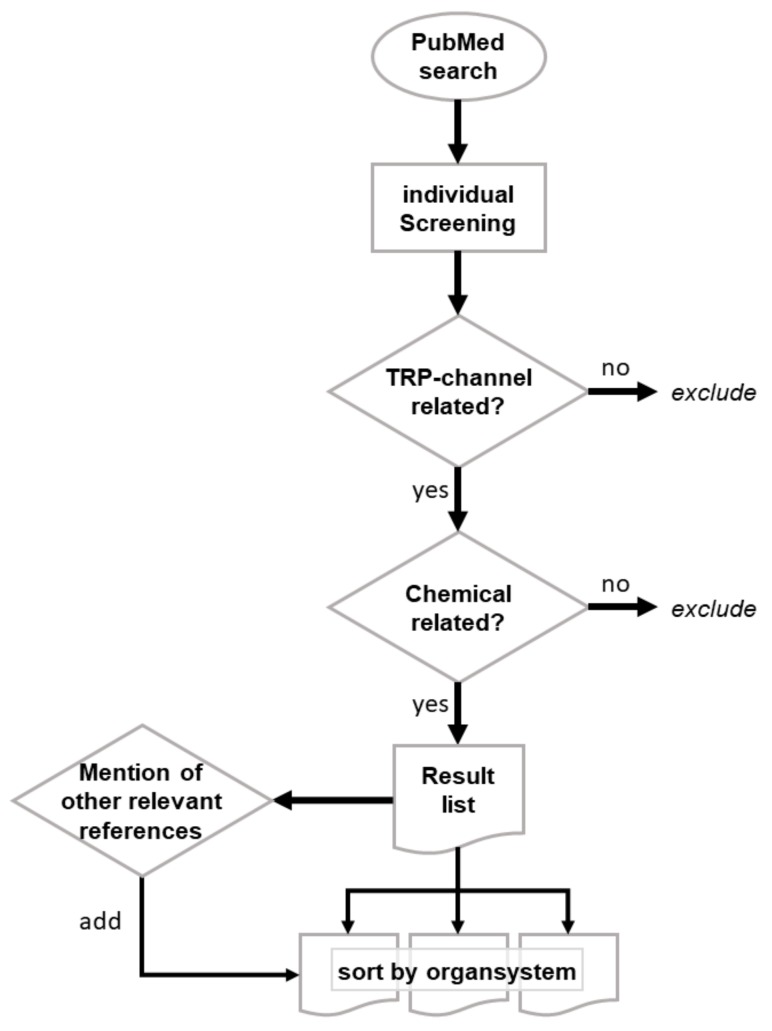
Search algorithm for the identification of relevant literature. The PubMed database was searched using “(trp channel) AND (toxic * OR chemical)” as a search term. The resulting 579 hits were screened. Literature not related to the topic of this review was excluded. If selected literature pointed to other references that were initially not identified in the PubMed search, these were also screened and included.

In this review we provide a synopsis of published literature dealing with the role of TRP channels in chemical toxicity. A NCBI PubMed search was performed using the search term “(trp channel) AND (toxic * OR chemical)”, which returned 579 hits. All abstracts were screened in detail by the authors according to the scheme provided in Figure 1. Finally, 139 publications were considered for this review and are listed in the reference section.

## 2. TRP Channels: General Structure and Function

**Figure 2:**
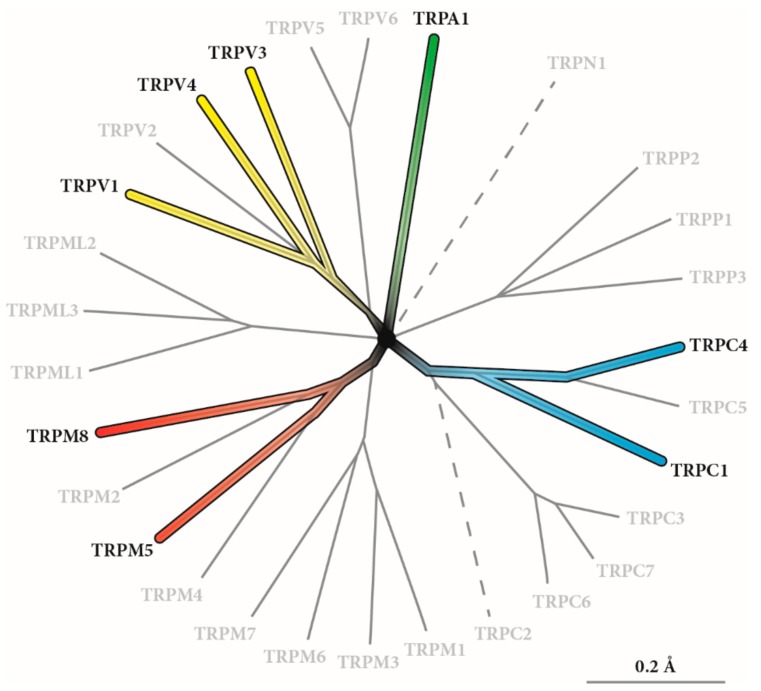
Phylogenetic tree of the transient receptor potential (TRP) channel family. In total, 28 human TRP channels have been identified so far. TRPN1 is expressed in insects and fish, but not in mammals, and TRPC2 is a pseudogene in humans. TRP channels that have been reported to be involved in chemosensing or that are affected by toxic chemicals are indicated by different colors ([20] modified).

TRP channels were first described in 1969 when Cosens and Manning noticed a distinct behavior of “a mutant strain of *Drosophila melanogaster* which, though behaving phototactically positive in a T-maze under low ambient light, is visually impaired and behaves as though blind” [12]. Electroretinograms identified the ion channel that was named after its electrophysiological function as transient receptor potential ion channel [13]. Montell and Rubin investigated the underlying biology in more detail using a *Drosophila* visual transduction mutant in which Ca^2+^-dependent adaptation to light was impaired [14]. The physiological function of the channel was further characterized by Minke in 1992 [15]. These milestones were the beginning of modern TRP channel research in mammals. Today, 28 mammalian TRP proteins are known, belonging to the TRP channel gene superfamily (Figure 2), and they represent one of the largest superfamilies of ion channels in the human genome [16,17,18,19,20]. The TRP channel family can be phylogenetically subdivided into six subspecies (TRPA1, TRPP, TRPC, TRPM, TRPML, and TRPV4 (Figure 2) [18,21], and its members are highly conserved between species, thus allowing a good translation of interspecies experiments in general [22].

**Figure 3:**
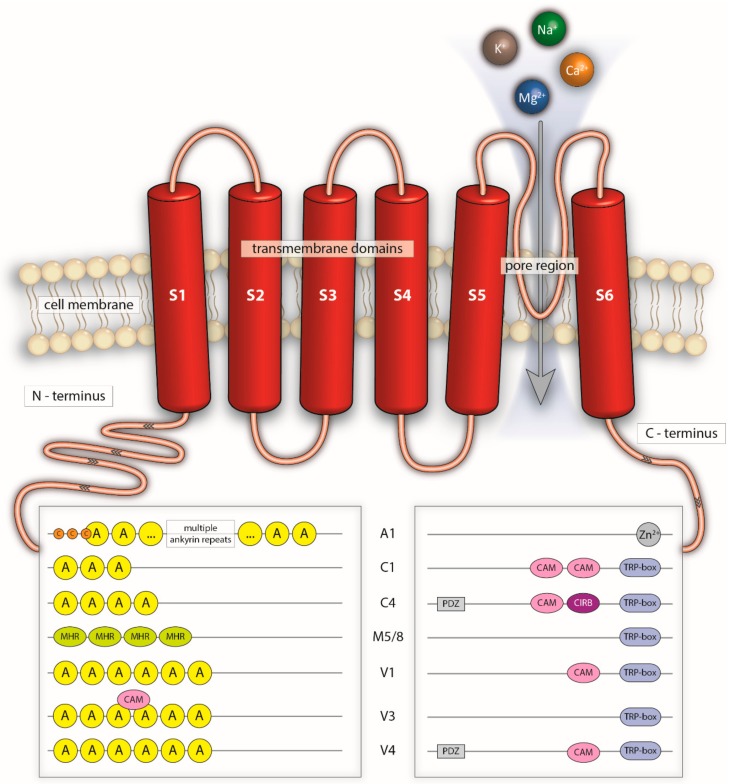
Schematic overview of TRP channel structure. “S1–S6” indicates the different segments of the transmembrane domains. “A” represents ankyrin repeats with reactive cysteines (“C”). “CAV1” is the caveolin interaction region, “MHR” represents the melastatin family channel homology region, “PDZ” symbolizes anchoring domain functions, “CAM” stands for calmodulin, “TRP box” is the motif containing the invariant EWKFAR sequence, “Zn^2+^” indicates a zinc-binding site, and “CIRB” is the calmodulin/IP_3_receptor-binding motif. The complete structure of some TRP channels are not known in detail. Synopsis from the following references: A1 [6,19,23,31,32], C1 [19,23,33], C4 [6,19,23], M5 [19,23,34], M8 [6,19,23,34,35], V1 [6,19,23,34], V3 [36,37,38,39], and V4 [6,19,23,34].

TRP channels form homo- or heterotetramers of single TRP proteins. Every protein consists of six helices, the transmembrane domains (TMs), which resemble the biggest part of the channel (Figure 3) [23]. TM 5 and 6 form the pore region, while the pore itself is semi-selective for the corresponding ion [23]. The function of helices 1 to 4 is not fully understood but seems to contain important channel modulatory binding moieties (e.g., the capsaicin-binding site is located in the TM2 and TM3 region) and is considered as the “sensor” part of the channel [24]. TRP channels are permeable to monovalent Na^+^, K^+^, and bivalent Ca^2+^ or Mg^2+^ cations [24]. Most members of the TRPV family are described as being rather nonselective in this context, with a preference of TRPV5 and TRPV6 for Ca^2+^ [25] while TRPM4 and TRPM5 are reported to be impermeable for Ca^2+^ [26,27]. The C- and N-termini are both located in the cytosol, and differ significantly between families, as they contain divergent binding motifs and reactive amino acids that modulate the channel function (e.g., the TRPA1 ligand allylisothiocyanate (AITC) has been described to activate the channel by binding to cysteine residues in the cytosolic ankyrin repeat sequence) [28] (Figure 3). However, it is important to note that also moieties at the C-terminal end and in the transmembrane parts themselves seem to have channel modulatory functions [29,30].

TRPA1 is the only member of the TRPA family, and it possesses multiple characteristic ankyrin repeat domains in the N-terminus. Other TRP channels, e.g., TRPV or TRPC, do also exhibit ankyrin repeat domains at their respective N-terminus, but to a lesser number compared to TRPA1. Another major difference is the lack of the so-called TRP-box, the sequence of the amino acids EWKFAR, which can be found in all TRP channels except TRPA1 [40]. TRPA1 is described to act as a chemo- or nociceptor [41]. The channel can be activated by pungent substances and plant ingredients like allicin from garlic, AITC from mustard oil, cinnamaldehyde from cinnamon oil, or gingerols from ginger [41,42,43]. Expression of TRPA1 is often found in neuronal structures, building heterotetramers with TRPV1 [44]. Here, TRPA1 is responsible for pain sensation, e.g., after consuming spicy mustard. In addition to the chemo-nociceptor function, TRPA1 was described to be sensitive to cold, which correlates well with the coexpression of TRPV1, as the latter is activated upon noxious heat [44,45,46].

The vanilloid TRP channel family (TRPV) consists of six members. TRPV1–4 show a closer phylogenetic relationship compared to TRPV5 and TRPV6 [20]. All TRPV channels contain ankyrin repeat domains at their N-terminus, as well as the TRP-box on the C-terminus. In contrast to other members of the family, TRPV1 carries a calmodulin-binding site at the C-terminus. TRPV channels can be activated by a plethora of different chemicals. TRPV1 is known to be sensitive to capsaicin or resiniferatoxin, and for some TRPA1 agonists, but to a lesser extent. Furthermore, the channel acts as a nociceptor for heat above 43 °C, and is involved in inflammatory pathways [47]. TRPV4, in contrast, is not known to be activated by plant-derived chemicals, although synthetic agonist like 4α-PDD or GSK1016790A were found to open the ion channel [48,49,50]. Functions of TRPV4 are related to controlling osmotic, chemical, and mechanical pressures, as well as sensing temperature [51].

Canonical TRP channels (TRPC) have also ankyrin repeat sequences at the N-terminus, and a TRP-box at the C-terminal site. As in TRPV channels, they contain a calmodulin-binding site [52]. In case of TRPC4, a PDZ binding site can be found downstream of the calmodulin binding site, which is lacking in the TRPC1 protein [52]. In general, all TRPC channels, with the exception of TRPC1, can be activated by phospholipase C stimulation via diacylglycerol [53,54].

Unlike TRPA, TRPV, or TRPC, TRPM channels do not possess ankyrin repeats at the N-terminus, but contain a distinct TRPM homology region [23]. A coiled-coil domain is located downstream of the TRP-specific TRP-box at the C-terminal end [55]. This coiled-coil domain is usually found in structural proteins like laminins, myosins, or fibrins. TRPM7 presents a kinase activity at its C-terminus, which is unique for that channel [56]. The physiological meaning is still not clearly understood, but is obviously related to mineral homeostasis [56].

## 3. Organ-Specific Expression of Chemosensing and Sensory TRP Channels

**Figure 4:**
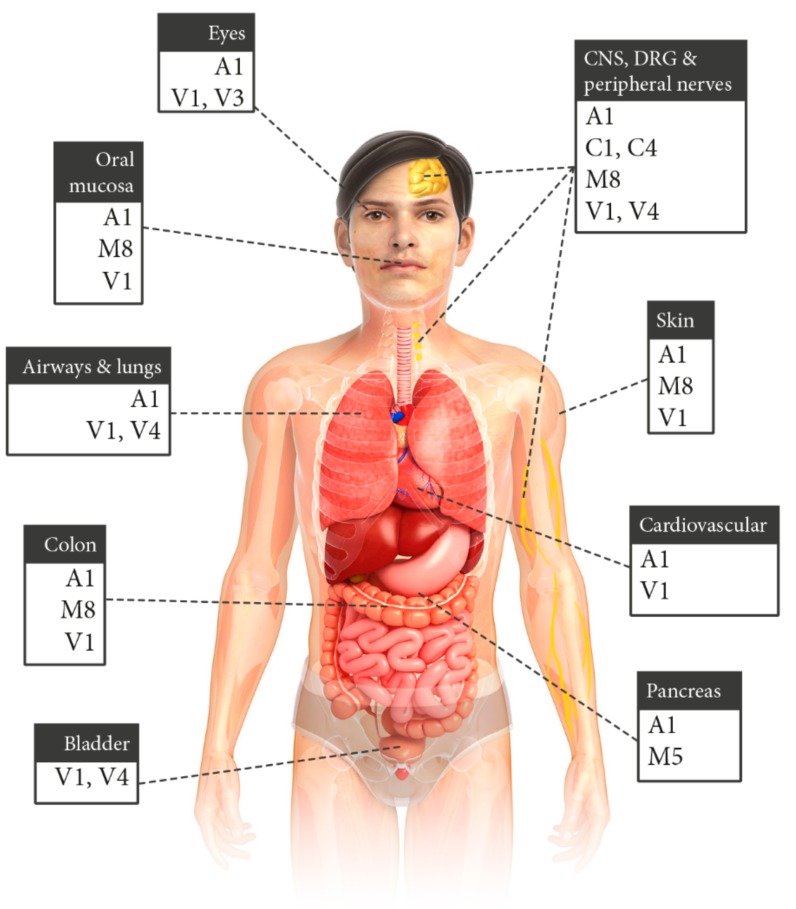
Schematic overview of chemical-sensitive or sensory TRP channel expression in different mammalian organs. Only TRP channels are illustrated that are discussed to be involved in chemical toxicity and are mentioned in this review.

**Figure 5:**
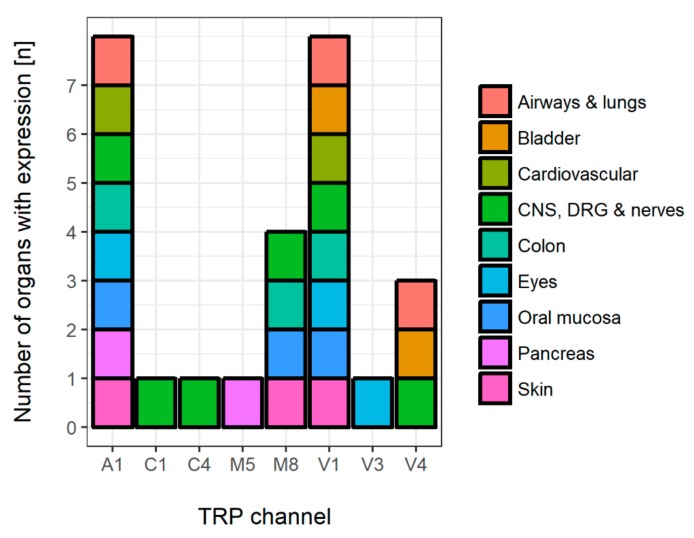
Overall numbers and subtypes of TRP channel expression with chemosensory properties. According to the considered literature in this review, TRPA1, TRPM8, and TRPV1 were identified as the most abundant channels. Colors represent organ-related expression.

TRP channels are ubiquitously expressed. While basic pharmacological channel functions are well-understood, many organ-specific functions are still elusive in mammals. Screening of the current literature revealed that TRPA1, TRPM, and TRPV seem to be important for chemical sensing or organ-specific responses to chemical compounds. TRPA1, TRPM8, and TRPV1 are the most frequently involved TRP channels in the considered organs regardless of the different cell types present in the respective tissue. They are followed by V4 while other TRP channels (C1, C4, M5, and V3) are of minor importance with regard to chemical sensing. Moreover, TRPA1, M8, and V1 are co-expressed in most organs. Figure 4 and Figure 5 summarize results of the screened literature. 

### 3.1. Neuronal TRP Channels

In principle, the human nervous system can be divided into two parts: (i) the central nervous system (CNS) consisting of the brain and spinal cord; and (ii) the peripheral nervous system (PNS), originating from the dorsal root ganglia (DRG) with the peripheral nerve trajectories. TRP channels play a major role in both parts. They are involved in sensing stimuli in the PNS, transferring signals via DRGs to the CNS, and regulating neuronal network function in the brain. In the respective tissues, TRPs can be activated by endogenous neurotransmitters and signaling molecules, e.g., diacylglycerol (DAG) or substance P, but also by mechanical stimuli like pressure or temperature, as well as chemicals from natural or synthetic origin [40,57,58].

#### 3.1.1. TRP Channels in the PNS and DRG

TRP channels were identified as key players in DRG, which link the peripheral nerve endings with the CNS. In DRG, TRPA1, and TRPV1 channels that are involved in nociception are found to be highly expressed, while TRPM8 or TRPV4 are expressed to a lesser extent. TRP channels do not serve exclusively as sensors of noxious stimuli. They are additionally affected by anesthetics, which are frequently used in clinical settings to minimize CNS function, or when locally applied, to block PNS signal initiation and transduction. TRP channels are highly expressed in all nervous tissue. Thus, it is not surprising that interaction of these channels with local anesthetics and central-acting narcotics influences analgesia and narcosis. A good example is the substantia gelantinosa, which plays a crucial role in pain reception. Here, neuronal cells respond with spontaneous L-glutamate release, which is mediated by TRPV1 and TRPA1 channels [59]. Moreover, induction of activating transcription factor 3 (ATF3), a reliable marker of nerve injury, was also found to rely on TRPV1 channels [60]. Wild type mice injected with capsaicin, mustard oil, formalin, or menthol into the hind-paw revealed increased ATF3 expression in distinct subpopulations of sensory neurons, whereas TRPV1 deficient mice did not [60]. Remarkably, only capsaicin-activated TRPV1 channels were required, while neither TRPA1, activated by mustard oil or formalin, nor TRPM8, activated by menthol or icilin, were necessary to increase ATF3 levels [60]. These findings clearly point to an essential role of especially TRPA1 and TRPV channels in nociception. Thus, it would be feasible to assume that analgesics and anesthetics should block these channels to prevent pain. However, some studies revealed that some general anesthetics surprisingly activate TRPA1 or TRPV1 channels. Eilers et al. found that inhalation of anesthetics with irritant properties indeed activate TRPA1, thereby inducing mechanical hyperalgesia and bronchial constriction [61]. Non-irritant anesthetics in contrast did not activate TRPA1, and provided no evidence for induction of hyperalgesia or bronchial constriction [61]. Other volatile anesthetics, i.e., isoflurane and the now obsolete chloroform, act as agonists of TRPV1 and TRPM8, while TRPA1 channels are inhibited [62]. These results are not completely in line with Eilers et al., who described increased sensitization of TRPA1 as a reason for hyperalgesia [62]. Lidocaine is another local anesthetic and anti-arrhythmic drug, which inhibits fast Na^+^-channels. Leffler et al. further described the activation of TRPV1, and in parts of TRPA1, by lidocaine [63]. Interestingly, the vanilloid binding domain is required for an activation by lidocaine, but not for sensitization of the channel as induced by other agonists [63]. To prove TRPV1 specificity, known antagonists like capsazepine or *N*-(4-t-butylphenyl)-4-(3-chloropyridin-2-yl) tetrahydro-pryazine-1(2*H*)-carboxamide (BCTC) were successfully applied in patch-clamp experiments using DRG or HEK293 cells [63]. Beside their narcotic and analgesic effects, anesthetics activate TRPA1, TRPV1 and TRPM8 channels, which are ubiquitous present in neuronal structures. Since the modulation of TRPV1 for analgesia seems very promising, investigations of new compounds are ongoing. Marwaha et al. examined niflumic acids as potential TRPV1 blockers to treat neuropathic pain [64]. For the experiments, they used an animal pain model in which stavudine induces TRPV1-mediated pain sensations. Here, TRPV1 protein expression in the CNS increased after stavudine exposure [64]. Remarkably, treatment with niflumic acid not only reduced pain, which was measured after different time points with different behavioral tests for mechanical hyperalgesia, tactile allodynia, motor coordination, heat and cold, but also reduced nitrosative stress [64].

Bang et al. have elucidated the mechanism of the anesthetic butamben, which has been used as a topical anesthetic since the early 1960s. It was suggested that voltage gated channels are inhibited by this compound, and this was confirmed by their study [65]. Additionally, butamben was shown to act as an inhibitor of TRPA1 as well as TRPV4, but not as an antagonist of TRPV1 [65]. 

Other naturally derived compounds may work in a similar way. Pellitorine, an extract of *Tradium daniellii*, was recently described as a new antagonist of TRPV1 [66]. A study by Olah and colleagues focused on pellitorine as a new anesthetic and pain killer. Although the chemical structure of pellitorine exhibited a relationship to capsaicin, a widely known TRPV1 agonist, the isolated pellitorine inhibited TRPV1 with an IC_50_ of 0.69 mM in TRPV1-transfected HaCaT cells after stimulation with 2 µM capsaicin [66].

Another substance found in species of *Artemisia*, *Blumea*, and *Kaempferia* is borneol. It was shown that this naturally derived compound antagonizes TRPA1 in a dose-dependent manner [67]. Borneol is already used in traditional medicine against bronchitis, rheumatic disease, or cell swelling [67].

Many other compounds, drugs, and chemicals are thought to interact with TRP channels. Based on TRPA1-induced hyperalgesia, Nozadze et al. investigated nonsteroidal anti-inflammatory drugs with regard to their potential to counteract TRPA1-induced side-effects [68]. In the in vivo study, diclofenac, ketorolac, and xefocam have proven to diminish AITC-induced TRPA1 activation, and hyperalgesia was mitigated [68].

Patients receiving anti-cancer treatment with oxiplatin frequently complain about neuropathy and hyperalgesia [69]. After treatment with oxaliplatin increased levels of cyclic adenosine monophosphate (cAMP) can be detected, which are able to sensitize TRPA1 as well as TRPV1 channels [69]. The study of Anand et al. measured increasing calcium responses after oxaliplatin treatment, which might cause hyperalgesia [69]. It is suggested that antagonist for TRPA1 and TRPV1 might be able to countermeasure oxaliplatin side effects [69]. A different study was able to confirm these results, as calcium signaling was altered after prolonged oxaliplatin treatment, and led to phosphoinositide-induced increases in intracellular calcium levels compared to the control group [70].

Paclitaxel, another chemotherapeutic drug, acts in a comparable manner. During chemotherapy patients suffer from neuropathic pain. It can be observed that protease-activated receptor 2 (PAR2, involved in inflammatory responses), as well as both kinases PKA and PKC are upregulated [71]. The upregulation of these proteins is described to sensitize TRPV1, TRPV4 as well as TRPA1 [71]. Thus, it is comprehensible that application of respective antagonists did attenuate observed pain responses in mice [71].

Pain relieving cannabinoids are currently in the spotlight of research. In case of the synthetic compounds *R*-(+)-(2,3-dihydro-5-methyl-3-[(4-morpholinyl)methyl]pyrol-[1,2,3-de]-1,4-benzoxazin-6-yl)-(1-naphthalenyl) methanone mesylate and (*R*,*S*)-3-(2-iodo-5-nitrobenzoyl)-1-(1-methyl-2-piperidinylmethyl)-1 hindole, it is reported that these substances activate TRPA1 and TRPV1 channels, but also desensitize these channels for other agonists like capsaicin or mustard oil [69,72].

Camphor, among other monoterpenes, is another agonist for TRPA1, which is like the above-mentioned cannabinoids, in that it effectively desensitizes the channel for more harmful activators [73].

In a comparable manner, it was shown that nitro-oleic acid desensitizes both TRPA1 and TRPV1. The inhibition was observed in cells expressing TRPA1 homomers, as well as TRPA1-TRPV1 heteromers [64]. Furthermore, pain reactions in test animals were counteracted by the substance and might be useful in a clinical environment to prevent unwanted TRPA1/TRPV1-induced hypersensitivities [64].

These studies have proven that TRP channels play a crucial role in the peripheral nervous system, as many anesthetics interact with TRP channels, whereas other substances, like many natural derived compounds, have a more beneficial effect on the body after interacting with a TRP channel. With increasing knowledge about pain signaling pathways, as well as inflammation, these ion channels can already be considered as promising pharmacological targets, yet many important crosstalks are still unknown and need further investigation.

#### 3.1.2. TRP Channels in the CNS and Cranial Nerves

Besides TRPA1 and TRPV1, which are known to be involved in pain sensation, TRPC, TRPV4, and TRPM8 are essential for signal transmission from the PNS or DRG to the CNS. Regarding physiological functions, TRPV4 channels are described to be involved in cell swelling and nociception [74]. TRPC1 and TRPC4 are expressed in the corticolimbic regions in the brain, controlling vasodilation and neurotransmitter release [75]. TRPC channels are also essential in the endothelial part of the blood-brain-barrier. Balbuena et al. reported upregulation of TRPC1 and TRPC4 after organophosphorus malathion/oxon or lead exposures in rats [76]. One major symptom that can be observed after malthion/oxon intoxication is the permeabilization of the blood-brain-barrier (BBB). Balbuena et al. reported increasing levels of TRPC1 and TRPC4 channels after exposure, which might lead to abnormal depletion of calcium stores. Subsequently, calcium-dependent pathways are likely to be dysregulated, contributing to the disruption of the BBB [76].

Comparable results were found by Zhang and colleagues, who blocked TRPC1 expression via RNA interference, and measured a decrease in lead levels (Pb) in the CNS [77]. In addition, overexpression of TRPC1 results in even higher levels of Pb in the CNS. Interestingly, knockdown of stromal interaction molecule 1 (STIM1), which is known as an endoplasmic reticulum Ca^2+^ sensor and together with Orai1 orchestrates the store-operated calcium entry, attenuated Pb-acetate entry [77].

Even though not directly related to chemical exposures, involvement of TRP channels in the pathophysiology of headache is discussed. In case of TRPV4, it is suggested that TRPV4 may lead to cell swelling in the brain, thereby causing headache due to extended pressure [74]. Treatment with arachidonic acid, among others found in caraway, or different phorbol ester compounds, agonize the channel, thereby reducing the pain [74]. Other compounds, e.g., umbellulone (UMB, which can be found in the leaves of the so-called “headache tree”) were also found to activate TRPA1 and TRPM8, thereby causing severe headaches and cold sensations [78,79]. Remarkably, UMB inhibited TRPA1 when applied in higher concentrations [79].

TRPA1 and TRPV1 are expressed in trigeminal nerves, and they may fulfill the role of sensing potential toxic substances or transmitting pain [80]. Despite the well-described activation of these channels by capsaicin, mustard oil, or allicin, a plethora of irritants affect these channels. Irritants like acetophenone, 2-ethylhexanol, hexyl isocyanate, isophorone, and trimethylcyclohexanol are channel agonists, and they were shown to decrease respiratory rates in vivo, which might be caused by direct interaction with TRPs [81,82].

Clotrimazole represents another potent agonist of those two channels in the trigeminal nerve [81,83]. Patients often complain about a burning sensation and irritated skin after topical treatment with the drugs. It was shown by Meseguer et al. that the activation of TRPV1 and TRPA1 might be responsible for this observed adverse effect [83].

Two studies of Kunkler et al. described the activation of TRPA1 as a cause for environmental-irritant-induced headaches [84,85]. Substances like formaldehyde or acrolein activated the ion channel, thereby releasing calcitonin gene-related peptide (CGRP) and substance P, which further resulted in neurogenic inflammation [84]. Additionally, it was reported that the meningeal blood flow increased after TRPA1 activation, which is in correlation to the increase of substance P, as the process can be inhibited by the application of CGRP [84].

In this context it is important to note that TRPA1 is most commonly co-expressed with TRPV1. Kunkler et al. further suggested that the therapy with resiniferatoxin might result in a decreased expression of TRPV1, which then may also attenuate co-expressed TRPA1 ion channels, thereby decreasing the amount of released CGRP and substance P. As a result, the blood flow should stabilize. Thus, resiniferatoxin might be a potential countermeasure against environmental irritant-induced headaches [84].

### 3.2. Upper Respiratory System, Airways and Lungs

Expression of TRPC, TRPM, TRPV, and TRPA1 has been described in the respiratory system. TRPA1 was found mainly in sensory nerves [10] and to some extent, also in the lung epithelia [86]. Stimulation of A549 cells, a human lung cancer epithelial cell line endogenously expressing TRPA1, with the TRPA1-specific agonist AITC, resulted in the increase of intracellular calcium ([Ca^2+^]_i_) and subsequent activation of MAP kinases [86], providing evidence for a functional role of TRPA1 in non-neuronal lung tissue. 

Eilers et al. reported that the pungent general anesthetic isoflurane is able to activate TRPA1 and induce TRPA1-mediated contraction of guinea pig bronchi [61]. In addition, desensitization of sensory nerves due to high concentration of the TRPV1 agonist capsaicin prevented isoflurane-induced bronchoconstriction. However, a TRPV1-specific antagonist alone had no effect on isoflurane-induced narrowing of the airways [61]. The authors proposed a TRPA1-dependent neurogenic mechanism for isoflurane-induced bronchoconstriction [61]. Animals also suffered from hyperalgesia after intraplantar injection of isoflurane, underlining the agonistic effect on TRPA1 channels [61]. These results are in line with the observed co-expression of TRPA1 and TRPV1 in a subset of C-fiber neurons [87]. Response of C-fibers towards chemical irritants was diminished in the nasal mucosa after pretreatment with capsaicin and/or AITC [88]. These findings point to a close interaction of TRPA1 and TRPV1 channels. In general, activation of neuronal TRPA1 and TRPV1 channels seems to initiate warning and defense reflexes, thereby attributing “protective” actions to the channel. In vitro experiments revealed that activation of TRPA1 by highly toxic alkylating compounds has a direct negative impact on cell viability [89]. This is in line with results published by Achanta et al., who suggested that TRPA1 and neurogenic inflammation contribute to the deleterious effects of alkylating compounds in vivo, activated either directly by alkylation, or indirectly, by reactive intermediates or pro-inflammatory mediators [90]. In contrast, zinc, a compound used in smoke screens, also activates TRPA1 [91] and induces lung cell injury [92], but not directly through TRPA1 [93]. Reactive oxygen species (ROS) as well as hypochlorite (OCl^−^), the oxidizing mediator of chlorine, activated Ca^2+^ influx and membrane currents in an oxidant-sensitive subpopulation of chemosensory neurons, which were identified as TRPA1-expressing cells [94]. TRPA1-mediated Ca^2+^ influx was also activated by chloramine-T, a widely used chlorine-releasing chemical disinfectant which is a strong respiratory irritant [94]. TRPA1-deficient (TRPA1^−/−^) mice were protected from OCl^−^-induced respiratory depression. In this study, response to other chemical stimuli remained unchanged in TRPA1^−/−^ mice, pointing to a certain selectivity of TRPA1 channels [94]. Toxic industrial isocyanates and tear gases, both known to cause severe pulmonary distress, were also found to activate TRPA1 channels [95]. Genetic ablation or pharmacological inhibition of TRPA1 dramatically reduced isocyanate- and tear gas-induced effects after both ocular and cutaneous exposures [95]. As demonstrated by Lehmann et al., in vivo sensory irritation due to 2-ethylhexanol, a potent human irritant, was dependent on both TRPA1 and TRPV1 channels [82]. Acrolein (2-propenal), a highly toxic and reactive compound present in tear gas, vehicle exhaust, and smoke from burning vegetation, including tobacco products, is also a very potent TRPA1 activator [96]. Responses were completely absent in cultures from TRPA1-deficient mice, demonstrating that TRPA1 is, indeed, an essential transducer of acrolein action [96]. Cells expressing TRPV1, TRPV2, or TRPM8 did not respond to acrolein [96]. Cigarette smoke-evoked cough was attenuated by a TRPA1-specific antagonist in guinea pigs [97] and unsaturated aldehydes in the cigarette smoke were identified as the main causative substances for TRPA1 activation [97].

Stimulation of airway epithelial cells with lipopolysaccharides (LPS), which is part of the wall of gram-negative bacteria and plays a crucial role in the immune response, resulted in an increase of [Ca^2+^]_i_ [98], which was found to be mediated by TRPV4 channels [98] that are abundantly expressed in these cells [99]. Upon LPS challenge, TRPV4-mediated ciliary beat frequently, and production of bactericidal nitric oxide increased, suggesting a protective role of TRPV4 [98]. In contrast to these findings, TRPV4 inhibition counteracts toxic lung edema and inflammation in vivo after chlorine exposures [100]. Here, chemical induced vascular leakage and airway hyperreactivity were suppressed, while blood oxygenation was significantly improved [100]. In vivo experiments conducted by Andres et al. point in the same direction: phosgene-induced pulmonary injury and lethality in mice were partially improved by post-exposure treatment with ruthenium red (RR), an unspecific TRP channel blocker [101]. Although the experimental design did not allow the identification of the distinct TRP channel, the authors speculate that TRPV4 may be one of the possible candidates.

Unspecific inhibition of TRP channels by intraperitoneal injection of RR attenuated adverse effects on cardiovascular function in vivo after pulmonary ultrafine nanoparticles exposures. Although the route of administration as well as the use of the broad and unspecific TRP channel inhibitor RR does not allow a precise correlation to a distinct TRP channel, the authors suggest a lung-nodose ganglia-regulated pathway via the activation of pulmonary TRP channels [102].

An involvement of TRPV1 channels in the pathophysiology of asthma triggered by inhalation of allergens and chemicals, was investigated by McGarvey et al. using pulmonary human tissue and cells derived thereof [103]. Remarkably, patients that did not respond to standard asthma therapy (i.e., corticosteroids) revealed a significant increase of bronchial TRPV1 expression compared to controls [103]. The authors suggest a role of TRPV1 channels especially in patients with severe, uncontrolled asthma. Both, TRPA1 and TRPV1, together with mast cells were found to play a major role in chemical-induced immune-mediated asthma after application of toluene-2,4-diisocyanate (TDI), a chemical with a large number of industrial applications [104]. Remarkably, TDI was unable to activate TRPV1 in that study, pointing again to a close cross-talk between TRPA1 and TRPV1.

### 3.3. Colon

Acute enteritis is a common and serious disease after food poisoning in human patients and animals. The immunological and microbiological aspects of the pathology of gastroenteric diseases including inflammatory bowel disease (IBD) are studied extensively [105,106]. However, the neural network is also known for its impact on observed hyperesthesia to various chemical stimuli. For instance, acetic acid and capsaicin were shown to provoke pelvic nerve activity in a rat model of inflammatory bowel disease induced by dextran sulfate sodium (DSS) [107]. The induced neural signaling by capsaicin was enhanced in rats with early stages of colitis, compared to healthy rats. The frequency of discharge was blocked by RR, an unspecific TRP channel inhibitor. It is assumed that the augmented nociception to TRPV1 agonists in the colitis model is associated with changes of inflammatory mediators that regulate the activity of nociceptors. One of these mediators is CGRP, a neuroinflammatory peptide which is expressed TRPV1-dependent in the colon. The expression of some, pro-inflammatory cytokines and chemokines like TNFα, IL-1, IL-6, CXCL10, MIP1 can be attenuated by treatment with icilin, a commonly used TRPM8 agonist [108]. TRPM8 is very prominent in human and mouse colon tissue. The expression of this TRP channel is upregulated in inflamed colon tissue from IBD patients, as well as in chemically induced colitis by DSS and 2,4,6-trinitrobenzenesulfonic acid (TNBS) in mice [108]. Administration of TRPM8 agonists in the colitis models inhibited TRPV1-dependent CGRP expression, reduced pro-inflammatory cytokines, and attenuated the common hallmarks of colitis such as bowel thickness and infiltration of granulocytes. These attenuating effects of icilin were not observed in TRPM8-deficient mice, which emphasizes the potency of TRPM8 as an anti-inflammatory therapeutic target.

The frequent contamination of wheat with the fungi *Fusarium* leads to food contamination by trichothecene mycotoxin deoxynivalenol (DON, vomitoxin) [109]. DON suppresses food intake and thereby affects energy balance, which is a major concern in human and animal health. The exocytosis of satiety hormones like cholecystokinin and peptide YY_3–36_ is induced by DON-activated calcium-sensing receptor (CaSR) and TRPA1-mediated Ca^2+^ entry into enteroendocrine cells [110]. Further analysis of the role of CaSR and TRPA1 in the observed anorexia and emesis will shed light on the underlying mechanisms, and can therefore serve as a model to understand comparable symptoms induced by other foodborne toxins, environmental toxicants and chemotherapeutic drugs [109].

### 3.4. Pancreas

Diabetes mellitus is one of the most common metabolic disorders in the industrialized western world. Both types of diabetes are diseases in which high blood sugar levels persist over a long time. Type II diabetes is characterized by the combination of insulin unresponsiveness in the target tissue and the failure of pancreatic beta cells to secrete sufficient insulin. Treatment options include glibenclamide, which blocks ATPase-sensitive K^+^ channels forcing beta cells to secrete more insulin. In addition, the TRPA1 channel was identified in pancreatic beta cells to transduce cationic non-selective currents. New studies revealed that the glibenclamide-induced intracellular currents are TRPA1-dependent [111]. However, the contribution of TRPA1 to diabetes treatment and the glibenclamide-related side effects like hyperactive bladder or abdominal pain is highly discussed [112]. TRPA1 seems to be a secretagogue which may still induce insulin secretion when cells do not effectively respond to glibenclamide after long-term treatment [111]. However, TRPA1 also disrupts beta cell physiology since long-term stimulation with TRPA1 agonists reduce both the responsiveness of insulin-secreting cells and the expression of pancreatic and duodenal homeobox 1 (PDX1), which is a transcription factor for insulin expression [112]. Consequently, the definite contribution of TRPA1 to glibenclamide-associated effects in type II diabetes patients remains to be clarified. Besides TRPA1, TRPM5 is also often associated with type II diabetes. Its expression in diabetic patients is negatively correlated with blood glucose concentrations [113]. TRPM5 is expressed in beta cells mediating depolarizing currents after glucose stimulation. Additional stimulation with stevioside, one of the main glycosides of the plant *Stevia rebaudiana* and used as a sweetener and sugar substitute, potentiates glucose-induced currents by increasing the frequency of Ca^2+^-oscillations, which triggers insulin response [114,115]. In wild type mice, treatment with stevioside reduced blood glucose significantly, but not in TRPM5-deficient mice. Moreover, stevioside protects mice on high fat diet from hyperglycemia. Therefore, stevioside, as TRPM5 modulator, is a promising therapeutic option in type II diabetes patients [114]. Chronic and acute pancreatitis have distinct histopathologies and etiologies, but are both accompanied by inflammatory events and pain [116]. Pain reflects the sensitization of pancreatic sensory neurons. The pancreatitis-associated pain was shown to be related to TRPA1 and TRPV1 expression, and it functioned in afferents in an experimental acute pancreatitis model in mouse [117]. The administration of specific TRP channel antagonists in early phases of recurrent bouts of acute pancreatitis significantly reduces inflammation. The combined antagonist treatment even ameliorates morphological changes in the pancreas [118]. Additional behavioral experiments demonstrated that both antagonists also attenuate pain and signs of discomfort. Therefore, targeting of TRPA1 and TRPV1 in patients with recurrent bouts of acute pancreatitis may inhibit the progression to chronic pancreatitis.

### 3.5. Bladder

TRP channels are widely expressed in the urinary tract, neuronal fibers innervating the bladder, and urethra and epithelial and mucosal layers of the bladder and urethral walls. Therefore, they are involved in many effects of toxicants, and are consequently an attractive pharmaceutical target for the treatment of (chemically-induced) disorders in the urinary tract.

It is reported that patients treated with cyclophosphamide suffer from cystitis accompanied by inflammation and bleeding. During treatment, the cyclophosphamide metabolite acrolein accumulates in the bladder and promotes inflammation [119]. In female Wistar rats with cyclophosphamide-induced bladder inflammation, a considerably high transcriptional plasticity of urinary bladder TRP channels were demonstrated. Chronic cystitis increased TRPA1 and TRPV4 transcripts in the (sub)urothelium of female rats, whereas in acute cystitis, TRPV1 and TRPV4 mRNA levels are decreased. However, TRPV1 and TRPV4 protein levels are increased in acute and chronic cystitis [120]. This lack of correlation between transcript and protein expression may reflect changes in posttranscriptional modifications. The observed TRPV1 increase is associated with bladder hyperreflexia [121]. In addition, antagonism of TRPV4 increased functional bladder capacity and reduced micturition frequency in mice and rats with acute cystitis, suggesting that chronic cystitis in human patients may improve with TRPV4 antagonist treatment [122].

In bladder transitional cell carcinoma TRPV1 expression declines with the increase of tumor grade [123]. Curcumin, a popular Indian food spice, is a very cytotoxic agent in bladder tumor cell lines. Since curcumin has a vanilloid structure with a vanilloid-like activity via a selective binding to TRPV1, TRPV1 activation by curcumin could be a possible mechanism to induce cell death and prevent tumor growth [124].

Patients with bladder overactivity are initially treated with *Botulinum* neurotoxin A (BoNT/A) to block the presynaptic release of acetylcholine from the efferent parasynapatic nerves, to paralyze the bladder temporarily [125]. However, BoNT/A also reduces TRPV1 expression on afferent nerves while the nerves remain intact. Therefore, BoNT/A affects bladder contractility by modulating the expression of TRPV1 on peripheral nerve fibers [124].

### 3.6. Skin

Phtalate esters are widely used as plasticizers for plastics, synthetic leather, vinyl flooring, wall coverings, paint, adhesive agents, and cosmetics. These esters are also commonly found in house dust and are associated with allergic diseases in children [126]. Dibutyl phthalate (DBP) directly activates TRPA1 in dorsal root ganglia isolated from mouse, as well as in cultured TRPA1-overexpressing cells [127,128]. Thereby both, DBP and also common TRPA1 agonists enhance skin sensitization to fluorescein isothiocyanate (FITC), which can be completely abrogated with the specific TRPA1 antagonist HC-030031. For TRPV1 a comparable contribution in such sensitization processes was shown, whereas TRPM8 is not involved in the enhancement of skin sensitization to FITC [129]. It is speculated that neuropeptides like CGRP are released from peripheral nerve endings via activation of TRPA1. In this context, it has already been described that CGRP triggers FITC-induced chronic hypersensitivity, while it suppresses trinitrobenzene-induced hypersensitivity. CGRP differently regulates the immunological contribution in form of T helper cell (T_H_1 and T_H_2) responses [130].

In cutaneous photosensitivity, it is widely accepted that the symptoms like pain and itching are based on the accumulation of porphyrins, which enhance the production of free radicals [131]. A common feature in patients suffering from porphyria or undergoing photodynamic therapy is the experience of strong pain from bright light. Classical photosensitizers like protoporphyrin IX, an intermediate in the heme biosynthesis pathway, transfer an electron to molecular oxygen-producing ROS. In photodynamic therapy, photosensitization is induced by excess levels of protoporphyrin IX, generating singlet oxygen to treat precancerous lesions [132]. The formation of ROS upon exposure to UV light, activates TRPA1 mediating the calcium entry into the cell. Mutation studies revealed that cysteine C633 and C651 are indispensable for disulfide bonds to mediate this UV-induced calcium influx. The activation of TRPA1 was abolished by the treatment with antioxidants acting as ROS scavenger, suggesting that oxidative processes are essential for UV-light-mediated TRPA1 activation. Protoporphyrin IX enhanced TRPA1 and TRPV1 activation. This activation in pain signaling nerve endings acts as cellular sensors to detect the oxidative stress produced by near-UV and visible blue light, which is enhanced in the presence of cellular or exogenous photosensitizers [131]. Therefore, selective antagonist may provide new therapeutic options for porphyria patients and for unlimited use of photodynamic therapy.

In the cosmetic industry chemical reagents are applied on the skin for rejuvenation. These procedures cause protein coagulation and tissue injury in various depth of the skin. Trichloroacetic acid (TCA), the most-widely used chemical peeler, exerts direct toxic effects on the skin. TRPV1 was indispensable for the expression of growth factors and cytokines after TCA treatment. TRPV1-deficient mice showed even more severe ulcerations compared to the wild type mice, suggesting that the modulation of TRPV1 can support rejuvenation treatments [133]. 

Clotrimazole (CLT) is a widely used antifungal for topical treatment of yeast infections of the skin, vagina, and mouth. CLT induced calcium influx in TRPV1-expressing cells, but not in closely related heat-activated TRPV2, TRPV3, and TRPV4 channels. In TRPV4-expressing cells, CLT even led to a reduction of basal calcium levels. HEK293 cells expressing TRPA1 clearly showed an intracellular calcium increase, though it was significantly slower than the influx in TRPV1 cells. In mouse experiments, the injection of CLT evokes noci-defensive behavior which could be attenuated by specific antagonist of TRPV1. Moreover, CLT shifts the voltage dependence towards a more negative voltage for TRPA1 and TRPV1, whereas for TRPM8 it shifts toward more positive voltages. Thus, CLT acts more as a gating modifier than a classical antagonist/agonist of these channels [83]. Due to its different effects on TRPA1 and TRPM8, CLT is a useful tool to discriminate between menthol-induced TRPA1 and TRPM8 responses in nociceptors, which are either potentiated by CLT in TRPA1-expressing cells or otherwise heavily repressed in TRPM8-expressing cells. CLT was identified as the most sensitive inhibitor of TRPM8 known so far. This opens up new possibilities as therapeutic agent against TRPM8-driven diseases like cold allodynia, and even certain types of malignancies [83].

Skin can itch for various reasons, including bacterial infections, insect bites, allergies, and different types of dermatitis. Itch is also a common side effect of drug treatment, such as in the case of the anti-malaria drug chloroquine (QC). In DRG a QC-induced calcium influx was shown to be TRP channel dependent. Knockout experiments in mice revealed that TRPA1 mediates the calcium flux, whereas TRPV1 was not involved [134]. In behavioral studies, wild type and TRPV1-deficient mice showed QC-induced scratching, whereas TRPA1^−/−^ mice did not, which is evidence that TRPA1-expressing neurons are required for QC-induced non-histaminic itch [134]. Also in acute contact dermatitis, resembled by an oxazolone-induced model of dermatitis in mice, TRPA1 is the determining channel to transduce the histamine-independent inflammation and pruritus [135].

### 3.7. Oral Mucosa

In oral mucosa TRPA1, TRPV1, and TRPM8 channels are predominantly expressed as chemosensors for irritants contained in food, drinks, and cigarette smoke. Lipophilic irritants such as mustard oil, capsaicin, and menthol activate TRPA1, TRPV1, and TRPM8, respectively, in sensory nerves of the buccal mucosa mediating the release of CGRP. Nicotine as a toxic substance, is, however, completely ineffective to induce CGRP exocytosis when applied in low concentrations [136]. Higher concentrations that may be reached in individuals consuming oral tobacco products evoked only a low CGRP secretion at pH 7.4. However, when deprotonated, uncharged and lipophilic nicotine was applied at pH 9, a robust TRPA1- and TRPV1-dependent response was observed. More physiological relevant experiments with full cigarette smoke containing hydrophilic nicotine, applied by smoking machines, showed moderate effects that were likely mediated by TRPA1 [136,137].

### 3.8. Cardiovascular System

There are only a few studies that suggest a role of TRP channels in chemical-induced cardiovascular toxicity. A single exposure to diesel exhausts was shown to increase the sensitivity of the heart to triggered arrhythmias [138]. This effect seemed to be mediated via the pulmonary activation of TRPA1, with subsequent sympathetic modulation, and it could be prevented by pre-incubation with RR [138]. Administration of TRPA1 or TRPV1 blockers prevented the increase of QRS duration, and a decrease in ST segment length (both are indicators for beginning cardiac stress), which were caused by diesel exhaust [138]. The results point to some evidence that activation of pulmonary sensory TRPA1 channels, which are known to be particularly sensitive to inhaled irritants, are involved in sympathetic activation [138]. Related results were found after pulmonary exposure of rats to ultrafine titanium dioxide particles, which resulted in elevated mean and diastolic blood pressure in response to norepinephrine [102]. RR inhibited substance P synthesis in nodose ganglia and associated functional and biological changes in the cardiovascular system [102].

### 3.9. Eyes

The eyes are one of the most sensitive organs with regard to irritation by chemicals. Most of the effects have been attributed to neuronal and sensory TRP channels (see section “Neuronal TRP channels”) like TRPA1. However, it was speculated that TRPV channels may play a role in a variety of cellular functions directly in the corneal epithelium [139]. TRPV1, V3, V4, and weakly V2 were detected by polymerase chain reaction (PCR) in a human corneal epithelial cell line [139]. Immunohistochemical stainings, together with PCR experiments and functional calcium measurements, revealed expression of TRPV3 in the murine cornea or primary corneal epithelial cells [139]. Carvacrol, the major component of plants such as oregano, savoy, clove and thyme is known as sensitizer and allergen, activates TRPV3 channels and directly affects cell viability [139]. Unfortunately, cytotoxicity after pre-incubation with TRP channel blockers and carvacrol exposure was not evaluated. Remarkably, low dose exposures with carvacrol, that did not result in a significant TRPV3 activation, were found to improve corneal wound healing in the scratch assay [139].

## 4. Closing Remarks

TRP channels are important actors in many physiological, but also pathophysiological processes. Over the last few years, they have come into focus as specific targets to counteract toxicity of certain chemical compounds. For some chemicals, e.g., acrolein, specific activation of TRP channels has been explicitly proven in vivo and in vitro, whereas for other compounds, the overall picture is less clear. As example for the latter, sulfur mustard has been clearly identified to activate TRPA1 channels in vitro [89]. However, contact to that agent seems not to induce acute pain or irritation symptoms. There are more examples pointing to a mismatch of in vitro results, and the clinical in vivo picture. Moreover, results obtained from rodent animal experiments do not necessarily reflect the human situation. This illustrates the need for further comprehensive investigations. Elucidation of the exact activation mechanisms, as well as the identification of the biological consequences of TRP channel activation through chemicals, should be in the focus of upcoming research.
